# The Interplay of Genetics and Environmental Factors in the Development of Obesity

**DOI:** 10.7759/cureus.1435

**Published:** 2017-07-06

**Authors:** Abu Baker Sheikh, Adeel Nasrullah, Shujaul Haq, Aisha Akhtar, Haider Ghazanfar, Amara Nasir, Rao M Afzal, Marvi M Bukhari, Ahtsham Yousaf Chaudhary, Syed W Naqvi

**Affiliations:** 1 Department of Internal Medicine, Shifa International Hospital; 2 Surgery, Texas Tech Health Sciences Center Lubbock; 3 Internal Medicine, Newark Beth Israel Medical Center; 4 Shifa College of Medicine, Shifa International Hospital; 5 Internal Medicine, Shifa College Of Medicine; 6 Shifa College Of Medicine

**Keywords:** genetics, environmental determinants of health, obesity, energy imbalance, genomic assay, epigenetics, biomarkers, epidemiology

## Abstract

Obesity is a major health issue in the developed nations, and it has been increasingly clear that both genetics and environment play an important role in determining if an individual will be obese or not. We reviewed the latest researches which were carried out to identify the obesity susceptible genes and to identify the metabolic pathways having a central role in energy balance. Obesity is a heritable disorder, and some of the many obesity susceptible genes are fat mass and obesity (FTO), leptin, and Melanocortin-4 receptor (MC4R). Glucose metabolism is the central pathway for fatty acid synthesis, de novo generating the major substrate acetyl-CoA. Further knowledge of these genes and their complex interaction with the environment will help devise individual, family and community-based preventive lifestyle interventions as well as nutritional and medical therapies.

## Introduction and background

**Figure 1:**
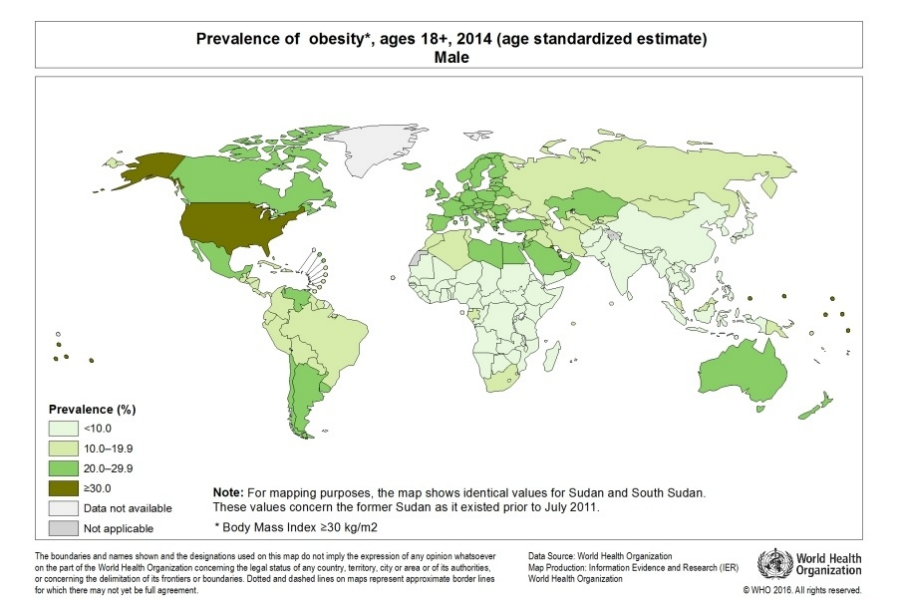
Prevalence of Obesity in Males with Age Greater Than 18 Years

**Figure 2:**
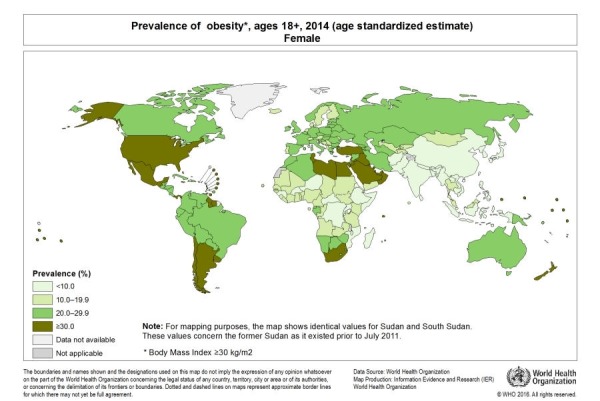
Prevalence of Obesity in Females with Age Greater Than 18 Years

Obesity is becoming an epidemic throughout the world, not only in the developed but also in the developing countries. It is increasing the health cost of the countries exponentially as a mode of interventions is required at all the levels. According to World Health Organization (WHO) in 2014, about 39% and 13% of the adults were found to be overweight and obese, respectively. The prevalence of obesity according to the gender has been shown in Figure 1 and Figure 2 [1-2].

Obesity is associated with various complications such as diabetes mellitus, hypertension and cardiovascular diseases [3]. Most of the complications are long-term with no cure and have a significant detrimental effect on the quality of life of the patient. Obesity is determined by the genetics as well as obesogenic environment [4-5]. It was first studied in 1994 that Ob gene and leptin play an important role in determining the body weight of an individual [6]. This study made the genetic contribution towards obesity exceedingly clear and set the platform to further assess the genetics and its relationship with obesity. There have been various studies, including those targeting twin and adopted family members, which showed that genetics may contribute towards obesity as much as 50-80% [7].

Obesogenic environment refers to the cheap and easy availability of high caloric diet and sedentary lifestyle. It has led to the positive energy balance and is possibly one of the major reasons for such high prevalence of obesity. Human eating habits can also be a part of the expression of different genes, and they can be one of the factors for developing obesity. Genetics and the environment and their complex interaction among themselves need to be looked upon in order to have a better understanding regarding obesity.

Here we review the genetics of obesity, which promises to show a better understanding of our current knowledge regarding inheritance and pathophysiology of obesity. Obesity susceptible genes and their interaction with the environment will also be reviewed. This is thought to be important as the increased knowledge of genetics and the obesogenic environment of the individual will help in planning better primary preventive measures at individual, family and community level. Not only this, but it may also help in therapeutic interventions and targeted medical therapy of the obese individuals.

## Review

Positive energy balance

Energy balance is the total amount of energy acquired from food and nutrition and the total amount utilized by the body. A body will assume a state of positive energy balance when the intake amount of energy will exceed the expenditure amount of the energy by the body. Similarly, a body will assume a state of negative energy when the amount of energy utilized by the body exceeds the amount ingested. Positive energy balance plays a vital role in determining overweight and obesity as it favors the storage of energy, which is in the form of fatty acids. These fatty acids stored in the adipose tissues may then be utilized if the body is deprived of energy. However, usually in an overweight or obese individual, there is a state of positive energy balance, and the body will continue to store the excess amount rather than utilize the already stored energy. Therefore, any genetic or environment component leading to a weight gain of the individual will affect the energy balance on the long-term basis.

Genetics of obesity

There is a strong association of genetics with obesity, but it is not the Mendelian way of inheritance. It means multiple genes are involved, and their complex interaction results in the manifestation of obesity. Increased research has led us from just a few obesity-associated genes back in 1994, to up to 50 loci. There can be a monogenic or a polygenic type of obesity. Monogenic obesity has been found to account for only 5% of the cases. In these cases, it has been well defined that genes coding for the proteins regulating the appetite center and satiety cause the pathological alterations and manifest most commonly as obesity [8]. Some of the monogenic obesity genes which manifest as severe obesity in children are leptin (LEP), leptin receptor (LEPR), pro-opiomelanocortin (POMC), and prohormone convertase 1 (PCSK1). Overall, 11 such genes have been identified that are associated with this type of obesity [8]. Polygenic obesity is studied through two main strategies, candidate genes studies, and genome-wide association studies.

Candidate Genes Studies

It is the study of genes of all the important metabolic pathways known to have an association with obesity. Major limitations of these studies include the smaller sample size and animal subjects as research candidates [9].

Genome-Wide Association Studies (GWAS)

It compares the genetics of a group of individuals having the trait under study (cases) and those who do not have the trait (control). By comparison of the genetics, the difference can be detected and analyzed. These studies are much more profitable and have a large sample size making the results more authentic [10-11].

Fat mass and obesity (FTO) gene

The FTO gene is a non-heme dioxygenase present on chromosome 16, which codes for the FTO protein. This protein is present in the nucleus and catalyzes the demethylation of nucleic acid bases associated with single-stranded DNA and RNA. GWAS study regarding body mass index (BMI) in 6148 subjects of Sardinia found a strong association between several FTO gene variants and BMI [12]. Another study comparing the affected homozygous subjects with the normal or heterozygous variants showed a strong association between FTO variant and increased BMI due to consumption of increased saturated fats after adjusting the total energy consumption of both the groups [13]. FTO polymorphism with obesity-related traits has also shown association with increased body weight [14-15], leptin levels [16], and waist circumference [17]. In this context, rs9939609 polymorphism is, up till now, the most studied FTO gene variant. Similarly, another study showed that the FTO variant children tended to consume more calories from fats as compared to the children who had homozygous normal trait [18]. There has been one study in Europe showing that the FTO gene variant does not interact with dietary factors, yet it is still associated with body mass and waist circumference. Further studies will be required to confirm that the FTO gene has an effect on the dietary factors to promote weight gain for obesity.

Melanocortin-4 receptor (MC4R) gene

MC4R gene encodes for MC4R protein and is a member of melanocortin receptor family present on chromosome 18q22. The MC4R is a hypothalamic receptor involved in energy homeostasis and its prime function is regulating food intake. Polymorphisms in the gene encoding this receptor are important for causing severe human obesity in both early and late onset forms of obesity. This protein interacts with the adrenocorticotropic and melanocyte-stimulating hormones (MSH). A study carried out in the murine model showed that the absence of the protein leads to obesity profile along with hyperphagia, hyperglycemia, and hyperinsulinemia as a consequence of uncontrolled overeating [19]. First MC4R variant was identified in 1998 [20-21], and since then approximately 100 different genetic variants have been described along with the functional diversity produced due to these variants [22]. Concerning interventional studies, one study did not find any association after nine months of intervention [23]; however, a recent study has shown that the weight loss intervention of the genetic variants showed a greater BMI decrease [24].

Obese (Ob) gene

This gene is present on chromosome 7q 31-33. The Ob-R gene is expressed ubiquitously, although expression of the different isoforms differs markedly between tissues. The physiologically essential Ob-R is the long form of the receptor (Ob-Rb) and is the dominant isoform in the hypothalamus [25]. Identification of Ob and Ob-R genes in the ob/ob and db/db mice has shown that a genetic approach can help in understanding some of the pathways that regulate body fat mass.

Metabolic pathways and environment

Functional integration of sterol regulatory element binding protein (SREBP), peroxisome proliferation activator receptor (PPAR) and other ligand binding transcriptional factors have an important role in oxidation reactions and fatty acid synthesis. The transcription factors that increase expression of genes involved in fatty acid β-oxidation are part of the nuclear receptor superfamily and include PPARα, PPARβ/δ, and retinoid X receptor (RXR) [26]. PPARα increases the expression of enzymes needed for the β-oxidation of fatty acids. Evidence suggesting that PPARα activators increase the β-oxidation of fatty acids and lead to decrease in body fatty acids is observed by a research carried out on mice. Mice fed on a high-fat diet along with fenofibrate weigh significantly less as compared to the mice that were not given fibrates. Fenofibrate is an antilipemic agent that is an agonist for PPARα which results in increased fatty acid oxidation, triglyceride elimination, an upregulation of lipoprotein lipase activity and very low-density lipoprotein (VLDL) catabolism. Also, mice with absent PPARα became obese with age as compared to the mice that had PPARα [27-28]. Medium-chain fatty acids (saturated and unsaturated) are the ligands for PPARα which stimulates the increased expression of genes encoding enzymes for β-oxidation of fatty acids in mitochondria. In brief, PPARα forms an obligatory dimer with RXR (which is activated by 9-cis retinoic acid) followed by this complex binding to the peroxisome proliferator receptor element (PPRE) within the promoter region of genes that encode enzymes that catalyze β-oxidation of fatty acids [26].

Dietary carbohydrates activate the transcription factors carbohydrate response element binding protein (ChREBP) and sterol regulatory element binding protein-1c (SREBP-1c) which are responsible for glycolysis and de novo fatty acid synthesis and storage in the adipose tissues when increased carbohydrates are being consumed [29-30]. Once the glucose levels are considerably high and they are being trapped inside the cells in the form of glucose-6-phosphate, this metabolite interacts with the low glucose inhibitory domain to prevent inhibition on Glucose Response Conserved element (GRACE), so that ChREBP enters the nucleus, dimerizes with max-like protein X (Mlx), and binds to the carbohydrate response element (ChoRE) in the promoter region of genes involved in fatty acid synthesis [30]. Both ChREBP: Mlx heterodimers and SREBP-1c induce genes encoding pyruvate kinase, acetyl-CoA carboxylase, fatty acid synthase, stearoyl-CoA desaturase 1, and glycerol-3-phosphate acyltransferase and interact at promoter regions whenever carbohydrate intake increases the glycogen storage capacity [31-32]. Polymorphism in SREBP-1c and Liver X receptors (LXR) has been found to be associated with obesity and morbid obesity [33].

Another area of research is the epigenetic inheritance of body weight that may occur through the interaction of environment-like nutrition. This is due to the dynamic alteration in transcriptional potential in the cells. For example, hypermethylation or hypomethylation of nucleotide bases, and histone modification via acetylation, methylation or phosphorylation are capable of modulating the transcription of genes involved in energy metabolism [34-35]. Nutritional status in prenatal and early postnatal life can alter the methylation molecules affecting many genes needed for metabolic and endocrine functions and lead to increased childhood obesity [36-37]. Epigenetic-environment interaction remains controversial as it is still not well understood whether epigenetics modification causes obesity or obesity alters the epigenetics in some way. As a result, epigenetics application in prevention and treatment is yet to be investigated [38-39].

Newer perspectives

One of the ways that the obesity genetics research could improve is through standardization of the variables which are taken into account. Some studies prefer BMI as the indicator while other studies take body fat mass or waist circumference, which results in a poor comparison between these studies. Therefore, a standard way to identify the genetic variation of obesity would prove beneficial. Epigenetics is a newer field of research and, as stated early on, it must be investigated upon further in order for it to be used as a target for primary prevention and therapeutic intervention. It has been shown that some of the genetic variation in the FTO gene is because of epigenetic changes.

## Conclusions

Various obesity susceptibility genes have been identified, but only a limited number of them have shown to have an interaction with the environmental components like altering the metabolic pathways of energy balance. Although the identification of the genes has been accomplished, the molecular basis of interaction of these genes with the environment will need much more time. Various study methods that have recently come into the light, like GWAS, will keep on demonstrating the genetic variants of the obesity susceptible genes. However, only 32 loci identified for BMI account for only 1.45% of the phenotypic variation. This suggests that further studies of epigenetic variation and the mechanism involved in obesity are needed. Also, the detection of genetically produced dysfunction by proteins such as MC4R which is involved in energy regulation, as a marker in diagnostic strategies, and as a predictor of remedies will be important for future management of obesity and might be a target for future gene therapy. Furthermore, it is believed that the increased knowledge and understanding of obesity susceptible genes and their complex interaction with the obesogenic environment will help in better individual, family, and community-based primary prevention and targeted nutritional and medicinal therapies.
